# Buffalo Medical and Surgical Association

**Published:** 1889-07

**Authors:** 


### Society Meetings.

Buffalo Medical and Surgical Association.—The last (June) meeting of the Association was largely attended by members and invited guests. A highly interesting discussion took place on The Best Medical and Surgical Treatment of the Insane. The leading participants in the discussion were Drs. Ring, Putnam, Hopkins, Crego, Hurd, and Stockton. We hope to publish their papers in full in the next number of the Journal. The next regular meetings will be held at the hall over No. 9 West Mohawk street, on the first Tuesday of every month at 8.15. Interesting programmes will be provided, and will be made up chiefly of clinical and pathological reports and the exhibition of cases, specimens, etc. The August meeting will be devoted to obstetric questions, three papers on important subjects having been promised for the occasion.

